# Mr. R. Alcock's Communication Respecting Baron Heurteloup's Instruments

**Published:** 1829-10-01

**Authors:** 


### LITHONTRITY.

Much attention has been excited lately by the consideration of the means by which calculi are broken in the bladder. This new operation, which many surgeons have laboured to improve with more or less success, appears to us of sufficient importance to make some account of it interesting to our readers. Having examined Baron Heurteloup's instruments, we gladly avail ourselves of the following general description of them forwarded to us by Mr. Rutherford Alcock. The character of that gentleman is sufficient guarantee for its accuracy.

"Prior to M. Heurteloup's improvements, the instrument by which the calculi were broken up, consisted of a tube, inclosing three branches, which on being forced out, opened by their own elasticity, and closed upon the stone by the act of drawing them in the tube. Within this tube also was a stylet with a drill at the end, which was made to perforate the stone by a rotatory motion communicated to it by a bow. Whilst the perforation of the stone was thus effected, the instrument was held in a kind of hand-vice which during the operation was confided to the hand of an assistant.

M. Heurteloup observing that this instrument only made one hole in the stone each time it was seized, concluded that this instrument was sufficient for small stones, the formation of one hole, enabling it to be broken up at once, but that it became insufficient in proportion as the stone increased in size—for the larger the calculus the more frequently it requires to be taken up in the bladder, in order to be broken up by successive perforations.

M. Heurteloup while he rendered full justice to this instrument by using it in cases of small stones, conceived and had constructed several instruments to fulfil the following indications which were not effected by the labours of those surgeons who had preceded him.

1st. *To seize a round or oval stone in the bladder one inch in diameter, and scooping it out, break it up at once.*

This indication was fulfilled by that which he calls the three-branch instrument with the *mandrin a virgule.**

In this instrument he retained the mechanism of the original three-branch instrument, and which was sufficient to take stones of this size, and re-placed the drill, which only made one hole of 3 lines and ½ in diameter with another, which by a single rotation made a cavity 10 lines in diameter. In this manner the center of a stone one inch in diameter, was immediately reduced to powder—and the remainder to a *shell or excavated fragments.*

2do. *To seize a stone of from* 12 *to* 18 *lines in diameter—excavate and break it at one sitting.* This he effected by a very ingenious apparatus which he calls *"evideur avec pince a forceps."*

---

* M. Heurteloup terms this head with the stem which supports it—"mandrin a virgule" because the small steel blade which is pushed out of the drill to make the excavation, is in the form of a virgule or comma (,).—R. A.

In this instrument the forceps destined to seize the stone is differently constructed to that previously mentioned containing three branches. It consists of four branches moveable separately or together, so that to grasp the stone, the surgeon takes it in a similar manner to the accoucheur when he employs the forceps. He only extends the last branch when the other three already retain it. The size of the calculus and its situation below the neck of the bladder, when it is of this size, have rendered this combination essentially necessary. This instrument has to assist it, in seizing the stone, another small pair of forceps which are very fine, the "*pince servante*" and which being introduced within the large forceps, is destined to place the body in the most favourable position for its destruction. The part of this instrument, destined to break it is composed of a perforator and evideur. The surgeon may obtain by means of this instrument, the excavation of a stone of 18 lines until its sides break down, and the fragments fall in excavated pieces.

The third indication M. Heurteloup wished to fulfil was—"*when the stones are reduced to shells or concave fragments, or when they are originally flat, to find the promptest and most effectual means of reducing them in sufficiently small morsels, and allow of their expulsion by the urine.*"

To solve this problem, M. Heurteloup invented the instrument he calls a "*brise coque.*"* Though it was possible to break the fragments with the three-branch instrument we first mentioned, he observed that its action was very slow, and that it had but little power over flat stones; he therefore saw the necessity of giving up the system of perforations to arrive at the desired result, and he substituted the action of two strong branches passing through a tube, and rubbed against each other by a mechanical apparatus, with the ends sufficiently large and powerful to crush and grind the body grasped by it; such is the effect of M. Heurteloup's brise coque. Its application is easy for the surgeon and unattended by pain to the patient. It is evident, on seeing the brise coque, that this instrument is capable of quickly destroying the calculi, and by no means liable to injure the bladder.

4*th. To place the patient and surgeon in the most favourable position for the action of the instruments, whichever they may be.*

The patient lying on a common bed during the search for the stone, and the instrument confided to the hand of an assistant, while he submitted to the action of the instruments, did not appear to M. Heurteloup to offer all the chances of success to the operator which he might easily command; he, therefore, invented a little bed, by which the patient was conveniently situated both as respected his position and the perfect facility with which the surgeon could act, and during the operation the instrument could be fixed, and constantly retained in the same place, making the chances of success much more numerous.

By this "*lit rectangle,*" then, as M. H. calls it, the surgeon is unconfined in his movements, and the instrument which grasps the stone is held steadily by a moveable vice fixing on to the frame, during the action of the drill, evideur, &c.

M. Heurteloup always employs this vice when he attacks calculi with his evideur—when he has only a small stone to destroy, and the three-branch instrument is sufficient, he employs it sometimes fixed, sometimes moveable."

Such is the able and interesting account of Baron Heurteloup's instruments, for which we are indebted to Mr. R. Alcock. We beg distinctly to state, that in thus taking notice of the Baron's instruments for lithontrity, we would not be thought for an instant to enter into any controversy between him and other gentlemen, nor to pronounce an opinion on their superiority or otherwise.

* It was for this instrument more particularly that M. Heurteloup received the prize of five thousand francs from the Institute of France.—R. A.

